# Cutaneous melanoma: a retrospective study of 18 years. Are there gender differences?^☆^^☆☆^

**DOI:** 10.1016/j.abd.2020.08.022

**Published:** 2021-07-18

**Authors:** Bruno de Castro e Souza, Diego Henrique Morais Silva, Neusa Yuriko Sakai Valente, Priscila Kakizaki, Maria Claudia Alves Luce, Luiza Groba Bandeira

**Affiliations:** Department of Dermatology, Hospital do Servidor Público Estadual de São Paulo, São Paulo, SP, Brazil

Dear Editor,

Although melanoma accounts for only 3% of malignant neoplasms of the skin, it accounts for the majority of deaths among skin tumors.^1^ Melanoma has a similar incidence in men and women up to 45 years of age. After that, the male sex predominates, so that after 75 years of age, the incidence in men becomes almost three-fold higher, raising the thesis that hormonal factors are implicated in its pathogenesis.^2^ Exposure to UV radiation, the number and characteristics of nevi and low phototypes are factors known to be associated with the development of melanoma, whereas the presence of ulceration and tumor thickness are associated with a worse outcome. Other risk factors and prognoses are controversial, including gender.^3^

An observational, descriptive and retrospective study of patients diagnosed with melanoma between 1998 and 2016 was conducted in a hospital in the city of São Paulo. The sociodemographic and clinical-pathological characteristics of these cases were collected from medical records aiming to describe the behavior of this neoplasm, with emphasis on the differences between sexes. The software used for data analysis was SPSS, version 25, and comparisons between the groups were performed using the Chi-Square test with correction for Fisher's^1^, ^2^, ^3^, ^4^, ^5^, ^6^, ^7^ exact test, when appropriate. The statistical significance was set at 5% (p < 0.05).

Over these 18 years, 359 melanomas were diagnosed; of these, 99 (27.6%) were characterized as melanoma *in situ* (mostly in female patients, with 201 cases; 57.3%) and 260 (72.4%) as invasive melanoma. Among the invasive melanomas, 152 occurred in female (58.5%) and 108 (41.5%) in male patients. Fig. 1 shows that most patients were diagnosed in the seventh decade of life (90 patients, 25.6%), a similar trend in both sexes. However, after the age of 70 years, the diagnosis of melanoma was proportionally higher in women. Fig. 2A shows that, over the years, there was a slight increase in the number of cases of melanoma, in both sexes. Despite this increase, a trend towards an earlier diagnosis of melanoma is observed, that is lesions with a lower Breslow index (Fig. 2B). Information on patient survival was found for 221 patients. Of these 178 (78.4%) survived the first five years after the diagnosis of melanoma.

**Figure 1:**
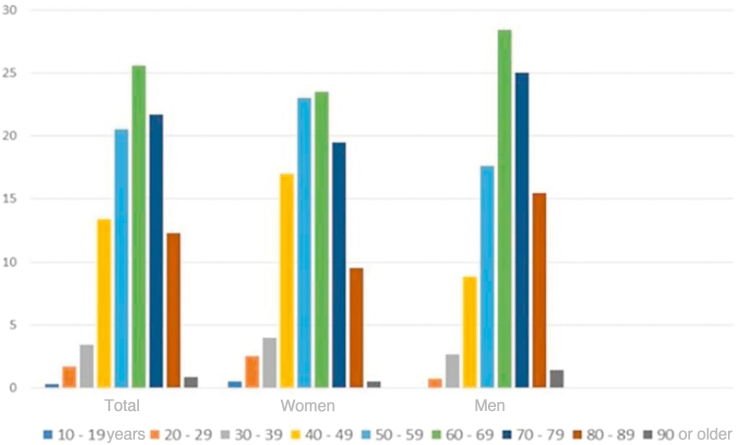
Cases of melanoma regarding the age at diagnosis (Total and differentiated by sex).

**Figure 2:**
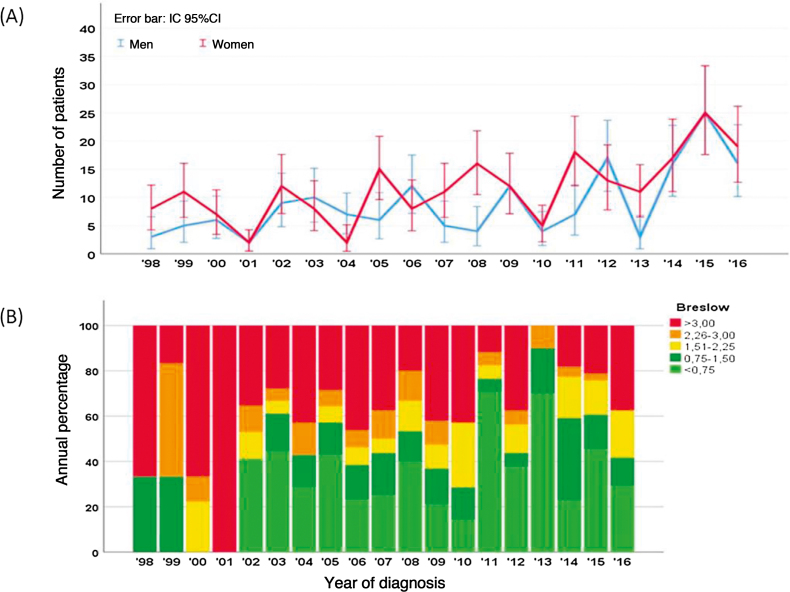
(A), Incidence of melanoma between 1998 and 2016 by sex; (B), Tumor thickness (Breslow) at diagnosis, with a decrease in the mean thickness in the last decade.

Table 1 shows the characteristics of invasive tumors, including differentiation by gender (male and female). As for the histopathological type, superficial spreading melanoma accounted for most cases, with 126 diagnoses (48.5%), followed by nodular melanoma in 58 (22.3%), acral lentiginous in 44 (16.9%), and lentigo maligna in 32 ( 12.3%). Men had a higher frequency of nodular melanoma 34 (13.1%) compared to female patients, 9.2% (p = 0.014). In women, the most common sites of melanomas were the upper limbs, 24.5% (51 cases). In contrast, in male patients, melanomas were found mainly on the head and neck, 31.4% (50 cases) and back region 23.9% (38 cases), with this difference between sexes being statistically significant (p = 0.001).

**Table 1:** Characteristics of invasive melanomas in the total sample and between sexes.

Number of invasive melanomas diagnosed		Total	Women	Men	p
		260 (100%)	152 (58,5%)	108 (41,5%)	
Histopathological type	Superficial spreading	126 (48.5)	79 (30.4)	47 (18.1)	0.014
	Lentigo maligna	32 (12.3)	22 (10.0)	10 (3.8)	
	Nodular	58 (22.3)	24 (9.2)	34 (13.1)	
	Acral lentiginous	44 (16.9)	27 (10.4)	17 (6.5)	
Location	Head and neck	90 (24.5)	40 (19.2)	50 (31.4)	0.001
	Anterior trunk	42 (11.4)	13 (6.3)	29 (18.2)	
	Back	78 (21.3)	40 (19.2)	38 (23.9)	
	Upper limbs	66 (18.0)	51 (24.5)	15 (9.4)	
	Lower limbs	40 (10.9)	31 (14.9)	9 (5.7)	
	Palmoplantar	53 (13.6)	31 (14.9)	18 (11.3)	
	Genitalia	2 (0.5)	2 (1.0)	0	
Clark	II	66 (24.7)	43 (28.9)	23 (19.5)	0.129
	III	69 (25.8)	42 (28.2)	27 (22.9)	
	IV	100 (37.5)	50 (33.6)	50 (42.4)	
	V	32 (12.0)	14 (6.9)	18 (15.3)	
Breslow	<0.75	94 (35.5)	60 (40.8)	34 (28.3)	0.156
	0.75 – 1.5	40 (15.1)	22 (15.0)	18 (15.3)	
	1.51 – 2.25	25 (6.8)	16 (10.9)	9(7.6)	
	2.26 – 3.0	22 (8.3)	10 (6.8)	12 (10.2)	
	3.00>	84 (31.7)	39 (26.5)	45 (38.1)	
Presence of mitoses		160 (52,8)	87 (49.7)	73 (56.3)	0.304
Presence of ulceration		66 (25.7)	33 (22.3)	33 (30.3)	0.148

As for the level of invasion, in female patients, most melanomas invaded only at the level of the papillary dermis - Clark II in 60 cases (40.8%). In contrast, most melanomas in male individuals showed Clark IV levels in 50 cases (42.4%); p = 0.129.

As for the Breslow index of melanomas in men, the majority had a tumor thickness > 3 mm (38.2%, 42 cases), while in women the majority had a Breslow index < 0.75 mm (58 cases, 41.4%); however, it was not statistically significant (p = 0.156). Mitoses in the invasive component of the melanoma were seen in 52.8% of cases. The presence of ulceration was reported in 22.3% of cases in women and 30.3% in men (p > 0.05). Although there was no statistically significant difference regarding the Breslow index, the presence of mitosis and ulceration, there was a difference in survival between the sexes. As shown in Fig. 3, 78.8% (119 cases) of women survived the first 5 years after the diagnosis, while in males this percentage was only 64.8% (70 cases); p = 0.012.

**Figure 3:**
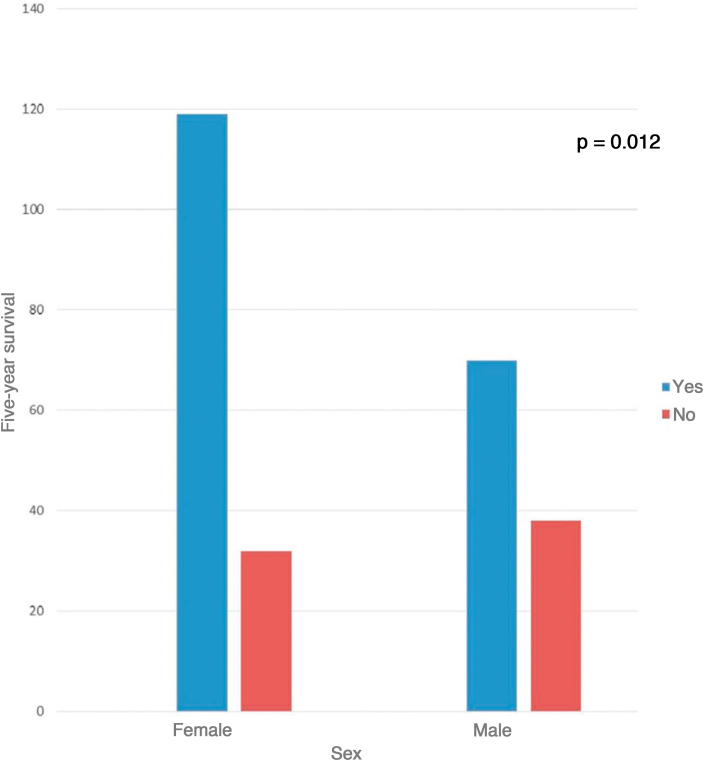
Five-year survival according to patient sex.

A similarly designed study was conducted in the same hospital analysing melanomas diagnosed between 1963 and 1997.^4^ During this period, 222 new cases of melanoma were identified, with an average incidence of 9.25 cases per year. In the present study, there was an average annual incidence of 19.94 cases. These data are compatible with epidemiological studies, which indicate an increase in the incidence of melanoma in recent decades. The present study identified differences between the sexes regarding the biological behavior and prognosis of melanoma. There are authors who consider sex as an independent prognostic factor for melanoma.^5^ Even after adjusting for variables that could result in bias (such as the histopathological type and age at diagnosis), the prognosis seems to be worse in males.^6^ One of the theories for explaining the better prognosis in female considers estrogen as a protective factor.^7^ However, the use of contraceptives or pregnancy don’t seem to alter women survival. In addition, studies have shown conflicting results regarding the survival of women in the post-menopausal period.

As the hormonal theory does not fully explain the better survival observed in women with melanoma, the oxidative stress theory looks promising. The increase in reactive oxygen species seems to be involved in melanoma carcinogenesis through several mechanisms: (a) DNA mutations; (b) cell proliferation stimulus; (c) inhibition of antigen-presenting cells. Comparatively, males have lower levels of antioxidants and, consequently, higher rates of oxidative damage.^8^

In summary, the present study found differences between sexes not only regarding the incidence of melanoma but also in the anatomical sites of the tumors, histopathological types, and in survival (greater in women). The authors believe that biological (hormonal influence, oxidative stress, and gene expression) and behavioral factors (exposure to UV light and self-care) work together to explain these differences.

## Financial support

None declared.

## Authors’ contributions

Bruno de Castro e Souza: Design and planning of the study; drafting and editing of the manuscript; collection, analysis, and interpretation of data; critical review of the literature.

Diego Henrique Morais Silva: Drafting and editing of the manuscript; critical review of the literature.

Neusa Yuriko Sakai Valente: Approval of the final version of the manuscript; effective participation in research orientation; intellectual participation in the propaedeutic and/or therapeutic conduct of the studied cases; critical review of the manuscript.

Priscila Kakizaki: Intellectual participation in the propaedeutic and/or therapeutic conduct of studied cases.

Maria Claudia Alves Luce: Critical review of the manuscript; drafting and editing of the manuscript.

Luiza Groba Bandeira: Critical review of the manuscript; drafting and editing of the manuscript.

## Conflicts of interest

None declared.

## References

[1] Leonardi G.C., Falzone L., Salemi R., Zanghì A., Spandidos D.A., Mccubrey J. (2018). Cutaneous Melanoma: From Pathogenesis to Therapy. Int J Oncol.

[2] Apalla Z., Lallas A., Sotiriou E., Lazaridou E., Iaonides D. (2017). Epidemiological trends in skin cancer. Dermatol Pract and Concept.

[3] Farahi J.M., Fazzari M., Braunberger T., Caravaglio J.V., Kretowicz A., Wells K. (2018). Gender diferences in melanoma prognostic fator. Dermatol Online J.

[4] Criado P.R., Vasconcellos C., Sittart J.A.S., Valente N.Y.S., Moura B.P.S., Barbosa G.L. (1999). Melanoma maligno cutâneo primário: estudo retrospectivo de 1963 a 1997 no Hospital do Servidor Público Estadual de São Paulo. Rev Ass Med Brasil.

[5] Joose A., van der Ploeg A.P.T., Haydu L.E., Nijsten T.E.C., de Vries E., Scolyer R.A. (2015). Sex diferences in melanoma survival are not related to mitotic rate of the primary tumor. Ann Surg Oncol.

[6] Crocetti E., Fancelli L., Manneschi G., Caldarella A., Pimpinelli N., Chiarugi A. (2016). Melanoma survival: sex does matter, but we do not know how. Eur J Cancer Prev.

[7] El Sharouni M.A., Witkamp A.J., Sigurdsson V., Van Diest P.J., Louwman M.W.J., Kukutsch N.A. (2019). Sex matters: men with melanoma have a worse prognosis than women. J Eur Acad Dermatol Venereol.

[8] Joosse A., de Vries E., van Eijck C.H., Eggermont A.M.M., Nijsten T., Coebergh J.W.W. (2010). Pigment Cell Melanoma Res.

